# Polyhydramnios at Term in Gestational Diabetes: Should We Be Concerned?

**DOI:** 10.3390/children12070920

**Published:** 2025-07-11

**Authors:** Mercedes Horcas-Martín, Tania Luque-Patiño, Claudia Usandizaga-Prat, Elena Díaz-Fernández, Victoria Melero-Jiménez, Luis Vázquez-Fonseca, Francisco Visiedo, José Román Broullón-Molanes, Rocío Quintero-Prado, Fernando Bugatto

**Affiliations:** 1 Area of Obstetrics and Gynaecology, Department of Child and Mother Health and Radiology, School of Medicine, University of Cádiz, 11009 Cádiz, Spain; merce.hormar@alum.uca.es (M.H.-M.); joseroman.broullon@uca.es (J.R.B.-M.); fernando.bugatto@uca.es (F.B.); 2 Division of Maternal-Fetal Medicine, Obstetrics and Gynecology Department, Puerta del Mar University Hospital, 11009 Cádiz, Spain; taniam.luque.spss@juntadeandalucia.es (T.L.-P.); claudia.usandizaga.sspa@juntadeandalucia.es (C.U.-P.); elena.diazfernandez@alum.uca.es (E.D.-F.); victoria.melero.sspa@juntadeandalucia.es (V.M.-J.); 3 Inflammation and Metabolic Syndrome in Pregnancy Group (CO25), Biomedical Research and Innovation Institute of Cádiz (INiBICA), 11009 Cádiz, Spain; luis.vazquez@inibica.es (L.V.-F.); francisco.visiedo@gm.uca.es (F.V.)

**Keywords:** gestational diabetes, polyhydramnios, amniotic fluid volume, adverse perinatal outcomes, adverse maternal outcomes, fetal overgrowth, macrosomia

## Abstract

**Background/Objectives:** Pregnancies complicated by idiopathic polyhydramnios are linked to a heightened risk of numerous maternal and perinatal complications. We aim to study the implications of polyhydramnios in term pregnancies complicated with gestational diabetes mellitus (GDM). **Methods:** A prospective cohort study including 340 GDM cases was conducted. An ultrasound scan was conducted at term between 37 and 40 weeks and amniotic fluid volume (AFV) was assessed by measuring the amniotic fluid index (AFI) and the single deepest pocket (SDP). Maternal demographics and obstetric and perinatal outcomes were evaluated after delivery. We performed comparisons between groups with normal AFV and polyhydramnios (AFI ≥ 24 cm or SDP ≥ 8 cm), and between groups with normal and increased AFV (AFI or SDP ≥ 75th centile). A multivariate logistic regression analysis was performed to study association between AVF measurements and adverse maternal and perinatal outcomes. **Results:** We found that women with GDM and polyhydramnios at term had a higher risk of maternal (54.3 vs. 27.5%, *p* < 0.001) and perinatal adverse outcomes (65.7% vs. 46.5%, *p* < 0.03). The increased AFV group showed a higher risk of fetal overgrowth (LGA: 21.4% vs. 8.2%, *p* < 0.001 and macrosomia: 19.8% vs. 5.4%, *p* < 0.001, respectively) and a lesser risk of delivering an SGA fetus (6.3% vs. 13.6%, respectively). Both AFI and SDP showed a significant correlation with newborn weight (r = 0.27; *p* < 0.001 and r = 0.28; *p* < 0.001, respectively) and newborn centile (r = 0.26; *p* < 0.001 and r = 0.26 for both). Subsequent to conducting a multivariate logistic regression analysis adjusted for pregestational BMI, nulliparity, and insulin treatment, both AFI and SDP were significantly associated with perinatal complications, but AFI showed a stronger association with fetal overgrowth (aOR 1.11; *p* = 0.004 for a LGA fetus and aOR 1.12; *p* = 0.002 for macrosomia) and with lower risk of delivering an SGA fetus (aOR 0.89; *p* = 0.009) or IUGR fetus (aOR 0.86; *p* = 0.03). ROC analysis showed a poor diagnostic performance of both AFI and SDP for identifying macrosomia (AUC 0.68 for AFI, and 0.65 for SDP). **Conclusions:** Detection of polyhydramnios at term, whether using AFI or SDP, identifies a subgroup of women with gestational diabetes with higher risks of obstetric and perinatal complications. Cases with increased AFV (AFI ≥ 18 cm or SDP ≥ 6.5 cm) are also associated with an increased risk of fetal overgrowth and may require more intensive monitoring for management and optimal delivery timing, with the aim of improve perinatal outcomes.

## 1. Introduction

Polyhydramnios, or hydramnios, denotes an excessive volume of amniotic fluid surrounding the fetus and affects 1–2% of pregnancies [1]. Prenatal diagnosis relies on the assessment of elevated amniotic fluid volume (AFV) by quantitative sonographic methods, including an amniotic fluid index (AFI) ≥ 24 cm or a single deepest pocket (SDP) of at least 8 cm [1,2,3,4]. While slight variations in these thresholds exist, they remain generally accepted. The AFI measurement involves summing the largest vertical pocket depth of amniotic fluid in each of the four uterine quadrants, while the SDP refers to the measurement of the amniotic fluid’s single deepest vertical pocket, free of the umbilical cord and fetal components. Since the AFI has a stronger predictive value and the SDP may cause overdiagnosis of high fluid volumes and additional interventions, some authors have suggested that the AFI may be a better predictor than the SDP for diagnosing high fluid volumes [5,6].

The implications of polyhydramnios can be significant for both maternal and fetal outcomes. Idiopathic polyhydramnios, defined as excessive amniotic fluid without an identifiable cause after thorough evaluation (excluding fetal anomalies associated with decreased or absent fetal swallowing, infections, and maternal diabetes), has significant implications for both maternal and fetal outcomes, including higher risks of preterm delivery, placental abruption, cesarean delivery, and postpartum hemorrhage [7,8,9]. Regarding adverse perinatal outcomes, polyhydramnios is linked to an increase in the incidence of intrauterine fetal demise, neonatal death, poor Apgar scores at 1 and 5 min, admission to the neonatal intensive care unit (NICU), macrosomia (birth weight ≥ 4000 g), and fetal malpresentation [9,10].

Maternal diabetes mellitus is the most frequent cause of polyhydramnios, accounting for 8 to 25% of cases. In women with gestational diabetes mellitus (GDM), the most probable explanation for the development of polyhydramnios is fetal polyuria secondary to maternal hyperglycemia. Elevated glucose levels in the mother’s blood cross the placenta, leading to fetal hyperglycemia and, consequently, an increase in urine production by the fetus, resulting in excessive amniotic fluid accumulation. This theory is supported by data showing a correlation between AFI and amniotic fluid glucose levels, positioning AFI as a possible indicator of glycemic management [11]. Since there are higher rates of polyhydramnios diagnosed by AFI and SDP for diabetic patients as opposed to those without, with these rates varying based on the type of gestational and pre-existing diabetes, monitoring amniotic fluid volume is an important aspect of managing pregnancies complicated by gestational diabetes.

However, it is crucial to remember that research specifically focused on polyhydramnios often exclude women with gestational diabetes to investigate the outcomes without the influence of this maternal metabolic condition. This underscores the importance of considering the maternal medical history, including the presence of gestational diabetes, when evaluating a case of polyhydramnios.

We aimed to understand if the diagnosis of polyhydramnios, frequently found in gestational diabetes at term, is associated with increased maternal and perinatal risks for these pregnancies.

## 2. Materials and Methods

### 2.1. Study Population

**Figure 1:**
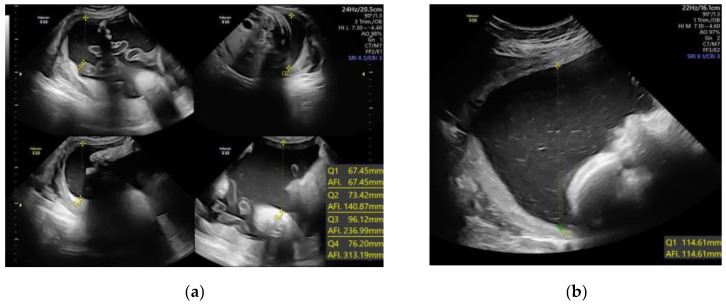
Amniotic fluid volume determination using quantitative sonographic techniques: (**a**) Amniotic fluid index (AFI); (**b**) single deepest pocket (SDP).

A total of 340 consecutive gestational diabetes cases were included in a prospective cohort study carried out at a tertiary referral university hospital during the years 2022–2024. Women with a singleton pregnancy and the diagnosis of gestational diabetes followed in our antenatal clinics between 37 and 40 weeks agreed to take part in the study after being informed of its focus. Exclusion criteria included multiple gestation, abnormal karyotype, fetal abnormalities, and maternal conditions such as infections, pregestational diabetes, or hypertension. At the initial prenatal appointment, height and pregestational weight were measured, and the pre-pregnancy BMI was determined. During the ultrasound scan, amniotic fluid volume was assessed semi-quantitatively in each case using the single deepest vertical pocket (SDP) and amniotic fluid index (AFI), according to the ISUOG Practice Guidelines [12], using a 2–9 MHz abdominal transducer on a VolusonTM S10 Expert ultrasound system (XdclearTM probe C2-9-D, GE Healthcare, Milwaukee, WI, USA). The following technique is used to measure AF pockets in accordance with the ISUOG guideline on the standard mid-trimester scan; maintain the ultrasound transducer on the mother’s abdomen at a perpendicular angle, measure the largest unobstructed amniotic fluid pocket that is at least 1 cm broad, clearly define the fluid pocket’s upper and lower margins, and use color Doppler to make sure the umbilical cord is absent from any pools of amniotic fluid where this is unclear (Figure 1).

In our study, polyhydramnios was defined as having either an AFI greater than or equal to 24 cm or an SDP greater than or equal to 8 cm. Increased amniotic fluid volume was defined as an AFI or SDP ≥ 75th centile; that is, an AFI ≥ 18 cm or SDP ≥ 6.5 cm.

GDM was diagnosed using the two-step approach and 100 g-OGTT, according to the National Diabetes Data Group (NDDG), with two or more values above 105, 190, 165, and 145 mg/dL after 0, 60, 120, and 180 min [13,14]. Birth weight centiles were obtained using customized birth weight criteria for a Spanish population, considering the gestational age at birth and the fetal sex [15]. When patients had an appointment in our antenatal clinic, women had an ultrasound scan by the obstetrician and a visit with the endocrinologist to check the blood glucose profile. Insulin treatment was commenced for patients who failed to meet the glycemic control objectives set for their respective groups regarding diet and exercise on more than three occasions within the same week. In our patients, we did not register data relative to blood glucose levels, as they are registered in the patient’s notebooks, and there is no laboratory test to check this, as there is, for example, in the case of pregestational diabetes with HbA1c. The pregnancy’s completion was arranged in compliance with the guidelines set by the Obstetrics and Gynaecology Service’s Unit. Labor was induced at week 41 if GDM was controlled with diet and exercise. Induction of labor took place at week 40 if GDM was managed with insulin-treatment. Obstetricians, midwives, and pediatricians’ clinical reports, which were entered into the Diraya Clinical Station’s computerized clinical records, provided information on gestational problems, deliveries, and newborns.

### 2.2. Diagnosis of Gestational Complications and Adverse Maternal Outcomes

The composite for gestational complications was defined as the development of at least one of the following adverse outcomes: gestational hypertension, preeclampsia, intrahepatic cholestasis, or cesarean delivery. The following criteria were used to identify hypertensive disorder of pregnancy: healthy, normotensive women without proteinuria or altered angiogenic ratio who had blood pressure readings of 140/90 mmHg at least twice at 4 h intervals following week 20 of pregnancy. The same blood pressure criteria were used to determine preeclampsia, together with the presence of proteinuria (≥300 mg per 24 h, as determined by 24 h urine collections) and/or an altered angiogenic ratio (sFlt-1/PlGF). Pregnancy-related intrahepatic cholestasis was identified when pruritus was linked to higher serum bile acids (values of more than 10 µmol/L) and/or aminotransferase levels.

The composite for adverse perinatal outcomes (APO) was defined as the development of any of the following complications: abnormal fetal growth, intrapartum fetal distress, Apgar < 7 at 5 min, umbilical cord pH < 7.20, NICU admission, shoulder dystocia, respiratory distress, hypoglycemia, hyperbilirubinemia, and intrauterine fetal death. Large for gestational age (LGA) and small for gestational age (SGA) neonates were defined by a birth weight >90th percentile or <10th percentile, respectively, based on growth curves tailored to the Spanish population by fetal sex [16]. IUGR was defined if the birthweight was <3rd percentile or abnormal Doppler was present. Newborns were classified as macrosomic if the baby weighed more than 4000 g at birth.

### 2.3. Statistical Analysis

Statistical analysis was performed using the SPSS 29.0 for Windows (SPSS, Inc., Chicago, IL, USA) computer statistics program. The Kolmogorov–Smirnov test and a histogram were used to examine the distributions. Depending on whether the variables had normal or nonnormal distributions, either Student’s t test or the Mann–Whitney U test (2-tailed) were used to compare two groups. For both parametric and nonparametric data, the Pearson or Spearman correlation coefficients were used to examine the correlations between the variables being studied. At the 95% level, statistical significance was established (*p* < 0.05). To assess the impact of AFI and DVP ultrasonography measurements on the composite of unfavorable pregnancy outcomes, multivariate analysis was carried out using logistic regression. The area under the curve (AUC), sensitivity, and false-positive rate for various ultrasound parameter cutoffs were all assessed using the ROC curve.

### 2.4. Ethical Considerations

The Good Clinical Practice (GCP) recommendations, European Directive 2001/20/CE, and ISO 14155 were all followed in this study, along with local laws. Under the number SICEIA-2024-002387, the local Ethical Committee (CE) examined and approved the final protocol and its modifications.

## 3. Results

### 3.1. Clinical and Anthropometric Characteristics

Table 1 displays the clinical and demographic details of the 340 pregnant GDM patients who were the subject of the study based on the occurrence of polyhydramnios (AFI ≥ 24 cm or SDP ≥ 8 cm) or increased amniotic fluid volume (AFI or SDP ≥ 75th centile; AFI ≥ 18 cm or SDP ≥ 6.5 cm). Statistically significant differences existed between the group with polyhydramnios (*n* = 35) or increased AFV complications (*n* = 116) and their respective normal AFV groups (*n* = 305 and *n* = 224) in terms of pregestational weight and BMI, newborn centile, and weight. Women with GDM and polyhydramnios or increased AFV had a higher pregestational BMI (30.3 and 29.3% vs. 27.9 kg/m^2^) and a higher mean newborn weight and centile (3515 g vs. 3271 g and 3538 g vs. 3242 g). The increased AFV group also showed a lesser proportion of nulliparity (37.9% vs. 56.7%) and a higher proportion of insulin treatment (47% vs. 35.7%).

### 3.2. AFV and Their Association with Adverse Maternal and Perinatal Outcomes

Compared to the normal AFV group, the polyhydramnios group had a higher chance of presenting with prenatal and postnatal complications (54.3 vs. 27.5%, *p* < 0.001 for maternal and 65.7% vs. 46.5%, *p* < 0.03, for perinatal complications, respectively). The increased AFV group showed a higher risk of fetal overgrowth (LGA: 21.4% vs. 8.2%, *p* < 0.001 and macrosomia: 19.8% vs. 5.4%, *p* < 0.001, respectively) and a lesser risk of having an SGA fetus (6.3% vs. 13.6%, respectively) (Table 2).

The most frequent maternal adverse outcome was delivery by cesarean section (51.4% and 27.6%, for polyhydramnios and increased AFV groups, respectively). The most frequent perinatal adverse outcomes were an LGA fetus (20% and 21.4%, for polyhydramnios and increased AFV groups, respectively) and macrosomia (14.3% and 19.8%, respectively).

The indications for NICU admissions in the polyhydramnios group (4) were one case of neonatal hypoglycemia, one case of respiratory distress, one case of infectious risk, and one case of fetal anemia. The indications for NICU admissions in the increased amniotic fluid group (11) were four cases of respiratory distress, three cases of neonatal hypoglycemia, one case of non-isoimmune hyperbilirubinemia, one case of infectious risk, one case of fetal anemia, and one case of neonatal acidemia (umbilical cord pH =< 7.0)

**Figure 2:**
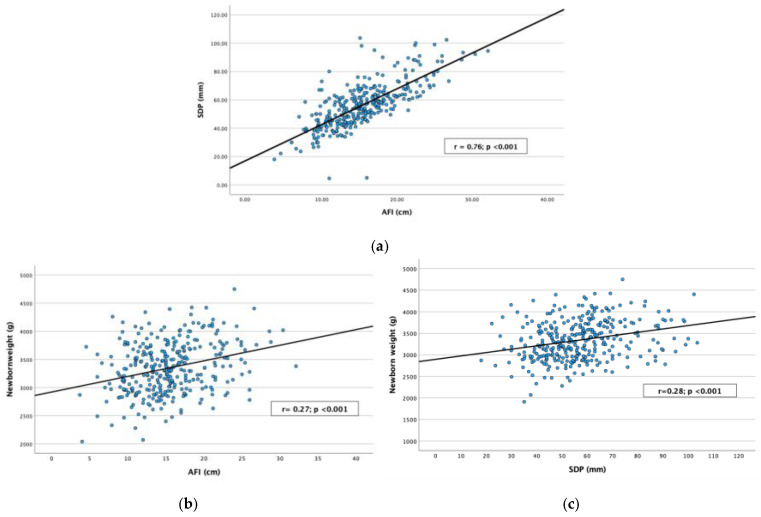
Scatterplot of amniotic fluid index (AFI, cm) in relation to single deepest pocket (SDP, mm) (**a**). Scatterplot of newborn weight (g) in relation to AFI (cm) (**b**) and SDP (mm) (**c**).

Ultrasound AFV measurements of AFI and SDP showed a significant correlation between them (r = 0.76; *p* < 0.001). AFI showed a significant correlation with newborn weight (r = 0.27; *p* < 0.001) and newborn centile (r = 0.26; *p* < 0.001) (Figure 2). A positive significant correlation between SDP and newborn weight (r = 0.28; *p* < 0.001) and newborn centile (r = 0.26; *p* < 0.001) was also found.

### 3.3. Logistic Regression Analysis

To find out how ultrasound AFV measurements (AFI and SDP) affected the composite for maternal and perinatal problems, multivariate analysis was carried out using logistic regression, with models adjusted for pregestational BMI, nulliparity, and insulin treatment (Table 3). Upon multivariate analysis, the adjusted OR of AFI and SDP measurements on the presence of maternal and adverse perinatal outcome were not statistically significant. The adjusted OR of AFI was 0.89 (95% CI, 0.82–0.97; *p* = 0.009) for the development of an SGA fetus and 0.86 (95% CI, 0.74–0.99) for an IUGR fetus. Additionally, the adjusted OR had a substantial impact on the growth of an LGA fetus, 1.11 (95% CI, 1.03–1.19; *p* = 0.004), and macrosomia, 1.12 (95% CI, 1.04–1.21; *p* = 0.002).

Regarding multivariate analysis for SDP, the adjusted ORs were statistically significant regarding the development of an SGA fetus, at 0.97 (95% CI, 0.95–0.99; *p* = 0.04); of a LGA fetus, at 1.03 (95% CI, 1.01–1.05; *p* = 0.009); and macrosomia, at 1.02 (95% CI, 1.001–1.04; *p* = 0.03). Finally, multivariate analysis showed an adjusted OR for low neonatal umbilical cord pH (<0.720) of 1.02 (95% CI, 1.003–1.04; *p* = 0.02).

### 3.4. Receiver Operating Characteristic (ROC) Curves Analysis

**Figure 3:**
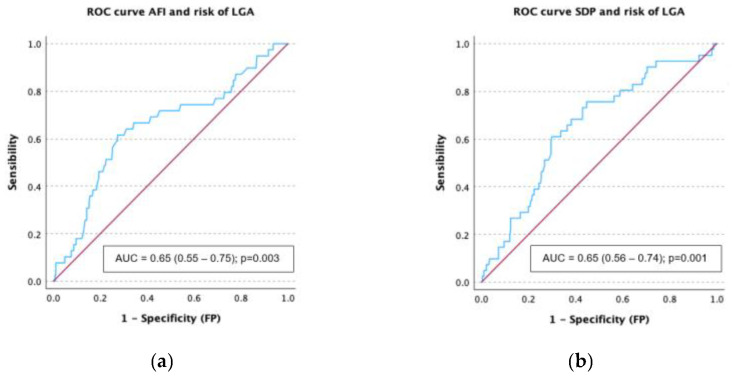

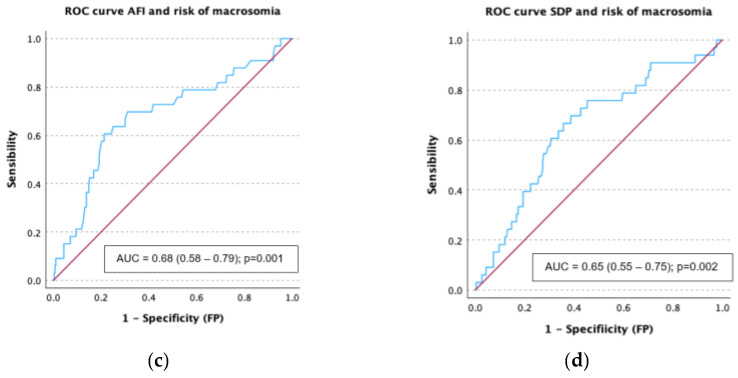
Receiver operating characteristic (ROC) curves for discrimination of the development of large for gestational age (LGA) by means of amniotic fluid index (AFI) (**a**) and single deepest pocket (SDP) (**b**) and for discrimination of the development of macrosomia by means of AFI (**c**) and SDP (**d**).

ROC curves were created to assess the diagnostic performance of AFV measurements regarding the development of macrosomia and LGA. For both AFI and SDP, the AUC for LGA detection was 0.65, with a 95% confidence interval of 0.55–0.75 and 0.57–0.74, respectively (Figure 3). Using a threshold of 17.1 cm for AFI yields a sensitivity of 61.5% and a specificity of 72.7% for detecting LGA, while a cutoff of 6.1 cm for the SDP results in a sensitivity of 61% and a specificity of 70.1%.

The AUC for identifying macrosomia was 0.68 (95% CI, 0.58–0.79) for AFI and 0.65 (95% CI, 0.55–0.75) for the SDP (Figure 3). While a cutoff of 17.95 cm for AFI produces a sensitivity of 60.6% and a specificity of 79%, a threshold of 6.1 cm for the SDP produces a sensitivity of 60% and a specificity of 70% for identifying a newborn weighting more than 4000 g.

## 4. Discussion

Our goal was to investigate the role of AFV in women with GDM at term and how it relates to unfavorable maternal and neonatal outcomes. Pregestational weight and body mass index were higher in women with polyhydramnios and increased amniotic fluid volume. Obesity is known to cause hormones to be out of balance, leading to chronic inflammation and increased insulin resistance [17], which leads to higher fasting glycaemia than in normal-weight women with GDM [18]. Maternal elevated glucose levels produce fetal hyperglycemia and may result in excessive amniotic fluid accumulation and the main cause of maternal-related polyhydramnios is maternal diabetes. In our cohort of GDM pregnancies with polyhydramnios, there was a higher risk of delivery via cesarean section. This could be explained, at least partially, by the higher rate of fetal malposition associated with an increased freedom for fetal movements inside the uterus. Ramos et al. [19] studied prediction models for primary cesarean sections in GDM-complicated pregnancies and found that polyhydramnios was an independent risk factor, almost tripling the risk.

Our results show that GDM cases with polyhydramnios at term are at increased risk of perinatal complications. In the same line, a recent systematic review and meta-analysis described that idiopathic polyhydramnios cases were at increased risk of perinatal complications [7]. One meta-analysis identifies polyhydramnios as 1 of 54 independent risk factors for placental abruption [20]; however, we did not report any cases, which may be explained by the fact that our studied population were all term pregnancies and this complication, as with preterm delivery itself, usually occurs before term and is mostly associated with the preterm premature rupture of membranes. Polyhydramnios has also been linked to higher neonatal weight [8,10], even in euglycemic pregnancies [21]. Interestingly, women with GDM in our cohort that presented increased amniotic fluid volume, defined as and AFI or SDP in the higher quartile (>75th centile), without reaching the level of polyhydramnios, also showed an increased risk of delivering an LGA fetus and macrosomia and a lower risk of delivering an SGA fetus, as has been described for polyhydramnios [10]. Concurrently, both AFV measurements, AFI and SDP, showed a significant correlation with newborn weight and centile (Figure 2). Given that cases of elevated amniotic fluid volume (AFI ≥ 18 cm or SDP ≥ 6.5 cm) correlate with a heightened risk of fetal macrosomia, enhanced surveillance may be needed for effective management and appropriate delivery timing, with the aim of improving perinatal outcomes.

In the multivariate analysis adjusted for pregestational BMI, nulliparity, and insulin treatment (Table 3), both AFI and the SDP were significantly associated with perinatal outcomes. Specifically, higher values of both AFI and SDP were positively associated with the delivery of LGA and macrosomic neonates and inversely associated with the delivery of SGA or intrauterine IUGR neonates. However, these associations were stronger for AFI than for SDP. Interestingly, in the case of AFI, our results imply that an increase of 1 cm would increase the risk of macrosomia by 12%. Similarly, Zou et al. demonstrated through logistic regression that AFI is an independent risk factor for macrosomia in women with gestational diabetes mellitus (GDM) [22]. This may be explained by the broader measurement range of AFI, which integrates multiple fluid pockets, compared to the more limited single-pocket approach used in SDP; so, when assessing a case with increased amniotic fluid, it may be preferable to measure the AFI, as has previously been proposed by other authors [3,6]. ROC curve analysis revealed that both AFI and SDP showed a similarly poor diagnostic performance for the identification of fetal overgrowth, with an AUC for LGA detection of 0.65. Although the AUC for identifying macrosomia was slightly better for AFI (0.68) than for SDP (0.65), any of them can be used as single markers for fetal overgrowth, but as a risk indicator within a comprehensive and multifactorial assessment, as we believe that other clinical and ultrasound factors such as maternal age, BMI, parity, obstetric history, glycemic control, insulin use, comorbidities, and fetal biometric parameters (including amniotic fluid, head circumference to abdominal circumference z score ratio, abdominal circumference z score, and estimated fetal weight) should be integrated to improve prediction, as suggested by other authors [23].

Conducting our study in term pregnancies (37–40 weeks) is particularly relevant, as this is the period during which key decisions about the management and timing of delivery are made to reduce risks associated with GDM. Evidence on planned birth at term in GDM is limited, but our study, by examining the association between term AFV and outcomes, contributes to the discussion on risk stratification in this population. Our analysis indicates that women with GDM who have an AFI greater than 18 cm or an SDP of more than 6 cm, are at a higher risk of producing a macrosomic child. Having an AFI or SDP nearer to the upper level of these limits in cases of gestational diabetes may be considered as a risk indicator within a comprehensive and multifactorial assessment and may indicate the need for attention by an experienced obstetrician.

Our study has the strength of being a prospective cohort study, which reduces certain biases inherent in retrospective designs. However, like most observational studies in this field, it may be subject to unmeasured residual confounding factors. Variations in the interpretation and technique of ultrasound measurements could also introduce variability, although standardized methods were followed. For future research, it is crucial to conduct larger prospective studies specifically focusing on the population of pregnancies with GDM and polyhydramnios to confirm these associations and determine the impact of different management strategies (e.g., timing and mode of delivery) on maternal and perinatal outcomes. It would be valuable to explore whether an AFV threshold exists, above which intensified fetal surveillance or intervention (such as induction of labor) improves outcomes in this specific group.

Regarding clinical implications, our findings suggest that the presence of polyhydramnios in a term GDM pregnancy should be considered an additional risk marker. This underscores the need for systematic assessment of amniotic fluid volume and, possibly, a multidisciplinary approach to the care of these pregnancies. Despite the limited high-quality evidence on the optimal management of term GDM in general, the identification of polyhydramnios could help guide clinical decisions.

## 5. Conclusions

Our results emphasize how crucial amniotic fluid volume measurement is for monitoring GDM pregnancies in the latter stages of pregnancy (polyhydramnios and particularly increased amniotic fluid volume) and that it should alert clinicians to a possible increased risk of complications such as cesarean section or macrosomia. This should prompt a reassessment of glycemic control, the closer monitoring of fetal well-being, and careful planning of the timing and route of delivery.

## Figures and Tables

**Table 1:** Maternal and newborn characteristics of the study’s participants.

	AFI ≥ 24 cm or SDP ≥ 8 cm (Standard Definition)			AFI ≥ 18 cm or SDP ≥ 6.5 cm (>75th Centile)		
	Normal AFV (*n* = 305)	Polyhydramnios (*n* = 35)	*p* Value	Normal AFV (*n* = 224)	Increased AFV (*n* = 116)	*p* Value
**Maternal** **characteristics**						
Maternal age (years)	34 ± 5.1	35.4 ± 5.9	*p* = 0.50	34.7 ± 5.1	35.2 ± 5.2	*p* = 0.42
Height (m)	1.62 ± 0.06	1.62 ± 0.06	*p* = 0.90	1.61 ± 0.06	1.62 ± 0.06	*p* = 0.23
Pregestational weight (Kg)	73.6 ± 17.2	79.9 ± 21.3	*p *= 0.04 *	72.3 ± 16.0	77.9 ± 20.4	*p *= 0.01 *
Pregestational BMI (Kg/m^2^)	27.9 ± 6.0	30.3 ± 7.4	*p *= 0.03 *	27.5 ± 5.8	29.3 ± 6.9	*p *= 0.01 *
Nulliparous (*n*, %)	155 (50.8)	18 (51.4)	*p* = 0.94	127 (56.7)	46 (37.9)	*p *= 0.003 *
Weight gain (kg)	7.9 ± 5.8	6.7 ± 6.3	*p* = 0.26	7.7 ± 5.9	7.9 ± 5.9	*p* = 0.80
Smoking (*n*, %)	42 (13.9)	7 (20.6)	*p* = 0.26	27 (12.2)	22 (19.3)	*p* = 0.08
Insulin treatment (*n*, %)	120 (39.5)	14 (40)	*p* = 0.95	80 (35.7)	54 (47)	*p *= 0.04 *
**AFV measurements**						
Gestational age at US scan	37 (37, 38)	38 (37,38)	*p* = 0.53	37 (37, 38)	37 (37, 38)	*p* = 0.28
AFI (cm)	14.5 ± 4.0	23.10 ± 4.4	*p *= 0.001 *	12.9 ± 3.0	20.1 ± 4.1	*p *= 0.001 *
SDP (cm)	5.3 ± 1.1	8.8 ± 7.9	*p *= 0.001 *	4.8 ± 0.9	7.3 ± 1.2	*p *= 0.001 *
**Newborn characteristics**						
Gestational age at birth (weeks)	39 (38, 40)	39 (39, 40)	*p* = 0.17	39 (38, 40)	39 (39, 40)	*p* = 0.84
Newborn male sex (*n*, %)	174 (57)	17 (48.6)	*p* = 0.33	123 (54.9)	68 (58.6)	*p* = 0.51
Newborn weight (g)	3271 ± 466	3515 ± 420	*p *= 0.02 *	3242 ± 439	3538 ± 452	*p *< 0.001 *
Newborn centile	49 (20, 80)	73 (36, 93)	*p *= 0.03 *	39 (14, 64)	75 (40, 94)	*p *< 0.001 *

* Statistical significance *p* < 0.05.

**Table 2:** Maternal and perinatal adverse outcomes according to the development of polyhydramnios (AFI ≥ 24 cm or SDP ≥ 8 cm) or increased amniotic fluid volume (AFI or SDP ≥ 75th centile; AFI ≥ 18 cm or SDP ≥ 6.5 cm).

	AFI ≥ 24 cm or SDP ≥ 8 cm (Standard Definition)			AFI ≥ 18 cm or SDP ≥ 6.5 cm (75th Centile)		
	Normal AFV (*n* = 305)	Polyhydramnios (*n* = 35)	*p* Value	Normal AFV (*n* = 224)	Increased AFV (*n* = 116)	*p* Value
**Gestational** **complications (** ***n*** **, %)**						
Composite for Gestational complications	84 (27.5)	19 (54.3)	*p *< 0.001 *	68 (30.4)	35 (30.2)	*p* = 0.97
Gestational hypertension	7 (2.3)	2 (5.7)	*p* = 0.23	6 (2.7)	3 (2.6)	*p* = 0.96
Preeclampsia	4 (1.23)	0 (0)	*p* = 0.98	4 (1.8)	0 (0)	*p* = 0.30
Hepatic cholestasis	2 (0.7)	0 (0)	*p* = 0.99	1 (0.4)	1 (0.9)	*p* = 0.56
Total cesarean deliveries	78 (25.6)	18 (51.4)	*p *= 0.001 *	64 (28.6)	32 (27.6)	*p* = 0.84
Urgent cesarean deliveries	62(20.3)	12 (34.3)	*p *= 0.05	49 (21.9)	25 (21.6)	*p* = 0.94
**Adverse perinatal outcomes (** ***n*** **, %)**						
Composite for APO	141 (46.5)	23 (65.7)	*p *= 0.03 *	102 (45.9)	62 (53.4)	*p* = 0.19
SGA (centile < 10th)	34 (11.1)	3 (8.8)	*p* = 0.99	30 (13.6)	7 (6.3)	*p *= 0.04 *
IUGR	15 (4.9)	1 (2.9)	*p* = 0.58	14 (6.3)	2 (1.7)	*p *= 0.06
LGA (centile > 90th)	35 (11.5)	7 (20)	*p* = 0.14	18 (8.2)	24 (21.4)	*p *< 0.001 *
Macrosomia (>4000 g)	30 (9.38)	5 (14.3)	*p* = 0.41	12 (5.4)	23 (19.8)	*p *< 0.001 *
Intrapartum fetal distress	23 (7.6)	4 (11.4)	*p* = 0.50	19 (8.5)	8 (6.9)	*p* = 0.60
Apgar < 7 at 5 min	3 (1)	0 (0)	*p* = 0.72	2 (0.9)	1 (0)	*p* = 0.72
Umbilical cord pH< 7.20	40 (7)	7 (20)	*p* = 0.26	29 (13)	18 (15.5)	*p* = 0.52
NICU Admission	25 (8.3)	4 (8.6)	*p* = 0.52	18 (8.1)	11 (9.5)	*p* = 0.65
Shoulder dystocia	2 (0.7)	2 (5.7)	*p *= 0.05	1 (0.4)	3 (2.6)	*p* = 0.11
Respiratory distress	5 (1.7)	1 (2.9)	*p* = 0.48	2 (0.9)	4 (3.4)	*p* = 0.10
Hypoglycemia (<40 mg/dL)	12 (3.9)	1 (2.9)	*p* = 0.60	10 (4.5)	3 (2.6)	*p* = 0.29
Hyperbilirubinemia (phototherapy)	13 (4.3)	0 (0)	*p* = 0.23	11 (5)	2 (1.7)	*p* = 0.11
IUFD	1 (0.3)	0 (0)	*p* = 0.89	1 (0.4)	0 (0)	*p* = 0.65

* Statistical significance *p* < 0.05.

**Table 3:** Multivariate logistic regression analyses for the composite of gestational complications, adverse neonatal outcomes, small for gestational age fetus (SGA, p < 10), intrauterine growth restriction (IUGR), large for gestational age fetus (LGA, p > 90) and macrosomia (>4000 g), neonatal intensive care unit (NICU) admission, and IUFD (intrauterine fetal demise): adjusted odds ratio (aOR, 95% confidence interval) for maternal pregestational body mass index (BMI), parity, and insulin treatment.

Variable	AFI (cm)		SDP (cm)	
	aOR (95% IC)	*p* Value	aOR (95% IC)	*p* Value
Composite of gestational complications	1.032 (0.98–1.087)	*p* = 0.22	1.010 (0.995–1.026)	*p* = 0.19
Gestational hypertension	0.947 (0.814–1.101)	*p* = 0.47	1.004 (0.964–1.045)	*p* = 0.85
Preeclampsia	0.969 (0.779–1.206)	*p* = 0.78	0.961 (0.891–1.036)	*p* = 0.29
Hepatic cholestasis	0.955 (0.710–1.283)	*p* = 0.75	1.010 (0.929–1.097)	*p* = 0.82
Cesarean delivery	1.036 (0.982–1.093)	*p* = 0.17	1.012 (0.996–1.028)	*p* = 0.15
Urgent cesarean delivery	1.041 (0.982–1.105)	*p* = 0.18	1.008 (0.991–1.026)	*p* = 0.35
Composite of APO	1.016 (0.971–1.064)	*p* = 0.50	1.009 (0.995–1.024)	p = 0.19
SGA (p < 10)	0.893 (0.821–0.973)	*p* = 0.009 *	0.975 (0.952–0.999)	*p* = 0.04 *
IUGR	0.860 (0.748–0.990)	*p* = 0.03 *	0.968 (0.932–1.005)	*p* = 0.08
LGA (p > 90)	1.110 (1.034–1.191)	*p* = 0.004 *	1.029 (1.007–1.051)	*p* = 0.009 *
Macrosomia (>4000 g)	1.127 (1.045–1.216)	*p* = 0.002 *	1.024 (1.001–1.047)	*p* = 0.03 *
Fetal Distress	0.974 (0.890–1.066)	*p* = 0.56	1.005 (0.980–1.031)	*p* = 0.68
Apgar < 7 at 5 min	1.087 (0.823–1.435)	*p* = 0.55	0.984 (0.912–1.062)	*p* = 0.67
Umbilical cord pH < 7.20	1.035 (0.968–1.107)	*p* = 0.31	1.022 (1.003–1.042)	*p* = 0.02 *
NICU Admission	0.998 (0.917–1.085)	*p* = 0.95	1.006 (0.981–1.031)	*p* = 0.64
Shoulder dystocia	1.122 (0.928–1.358)	*p* = 0.23	1.025 (0.968–1.085)	*p* = 0.40
Respiratory distress	1.121 (0.960–1.309)	*p* = 0.15	1.015 (0.971–1.062)	*p* = 0.51
Hypoglycemia (<40 mg/dL)	0.910 (0.799–1.038)	*p* = 0.16	0.983 (0.946–1.022)	*p* = 0.38
Hyperbilirubinemia (phototherapy)	0.959 (0.843–1.091)	*p* = 0.52	0.971 (0.931–1.012)	*p* = 0.16
IUFD	0.696 (0.363–1.335)	*p* = 0.27	0.931 (0.788–1.099)	*p* = 0.39

* Statistical significance *p* < 0.05.

## Data Availability

The original contributions presented in this study are included in the article. Further inquiries can be directed to the corresponding author.

## Abbreviations

The following abbreviations are used in this manuscript:

AFV	Amniotic Fluid Volume
AFI	Amniotic Fluid Index
APO	Adverse Perinatal Outcomes
BMI	Body Mass Index (kg/m^2^)
GDM	Gestational Diabetes Mellitus
LGA	Large for Gestational Age fetus
SDP	Single Deepest Pocket
SGA	Small for Gestational Age fetus
IUFD	Intrauterine Fetal Demise
IUGR	Intrauterine Growth Restricted Fetus
